# Supplementary material for: Perception and Understanding of Guideline Daily Amount and Warning Labeling among Mexican Adults during the Law Modification Period
Source: Nutrients. 2022 Aug 18;14(16):3403. doi: 10.3390/nu14163403 (PMC9415154; doi:10.3390/nu14163403)

Figure 1. Products used in the study

1. First evaluation

1a. Salty snacks

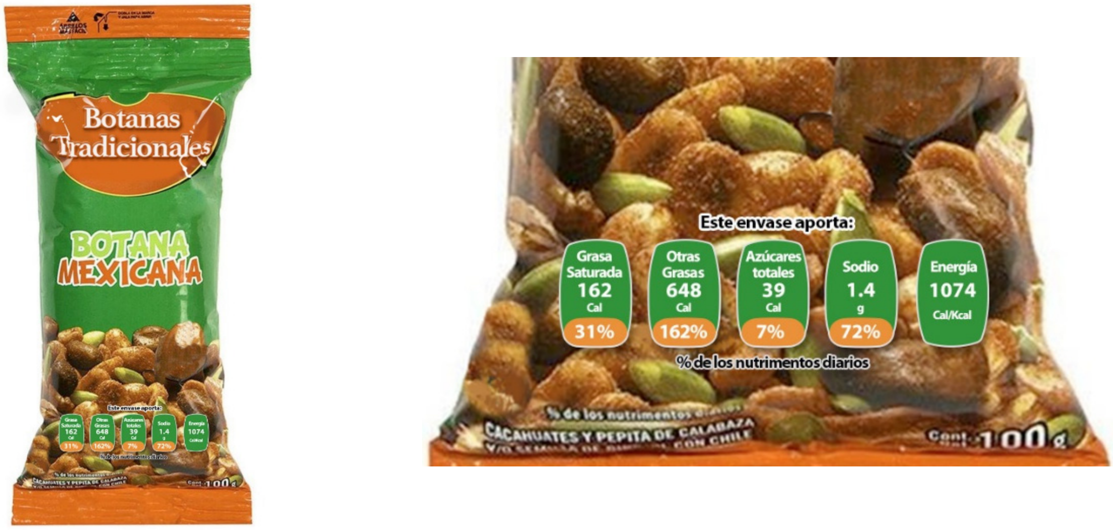

1b. Ready-to-eat cereal

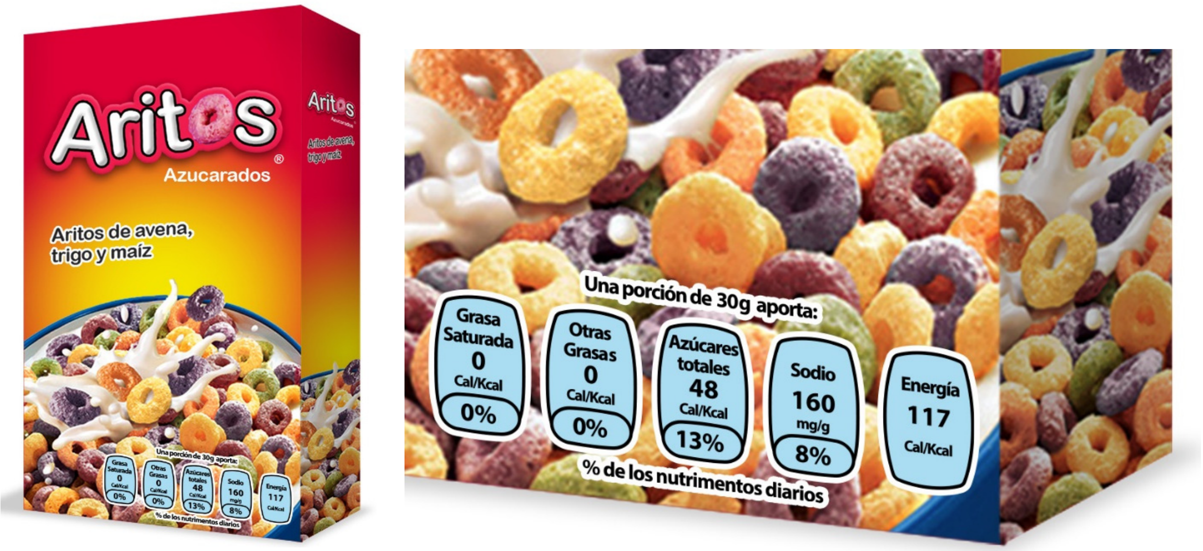

1c. Sugar Sweet Beverage

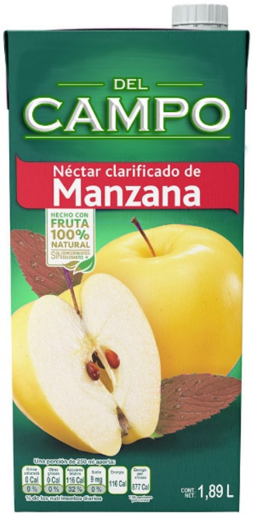

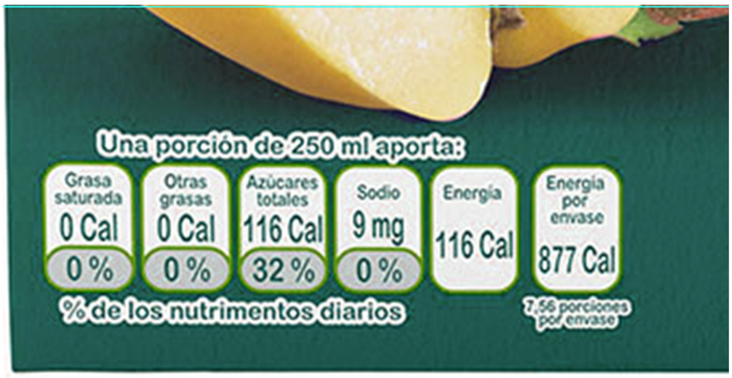

1d. Dairy

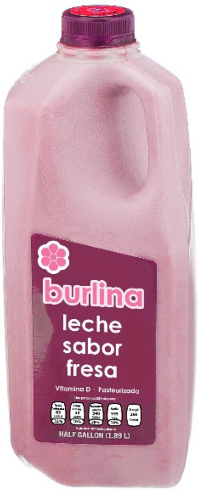

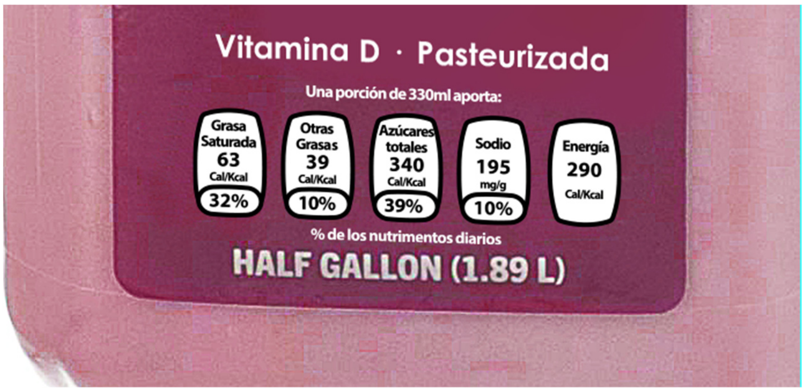

1e. Ready-to-eat Food

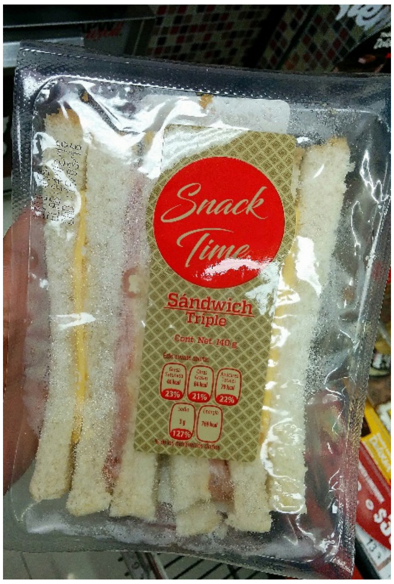

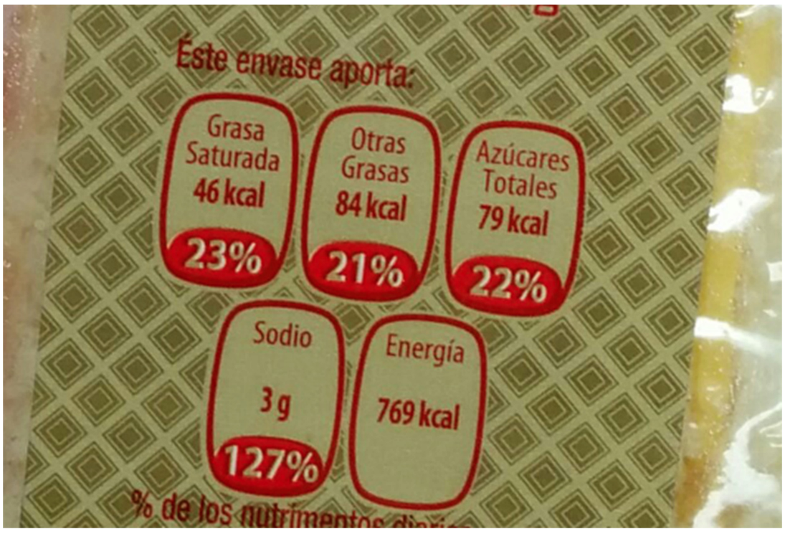

2. Second Evaluation

2a. Salty snacks

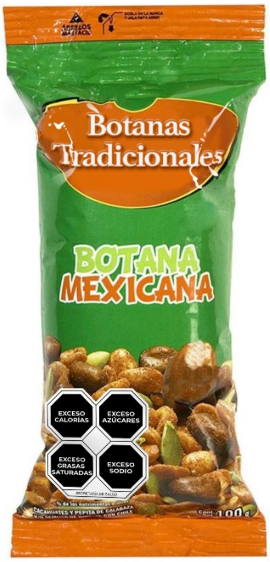

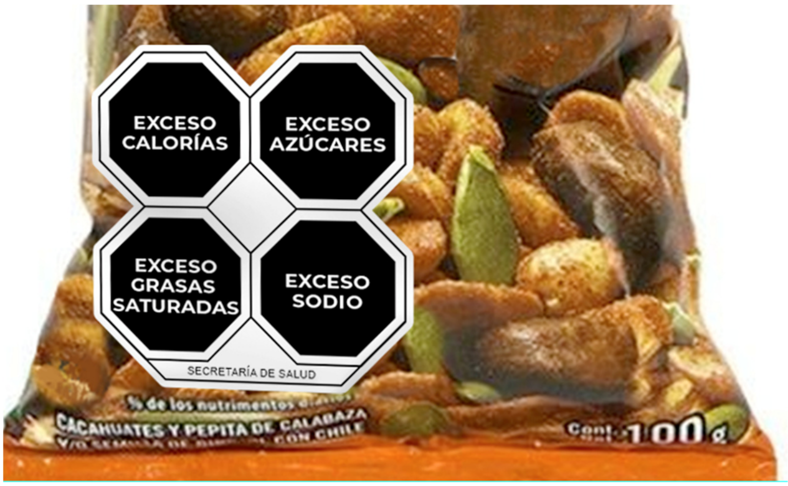

2b. Ready-to-eat cereal

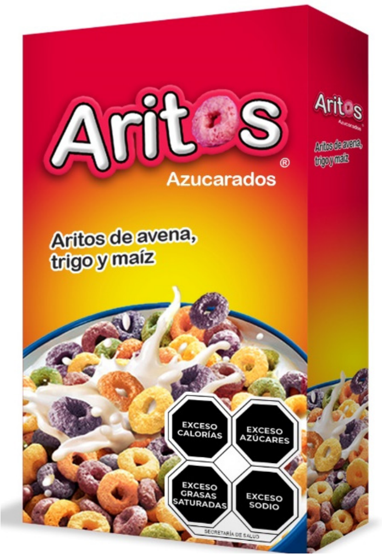

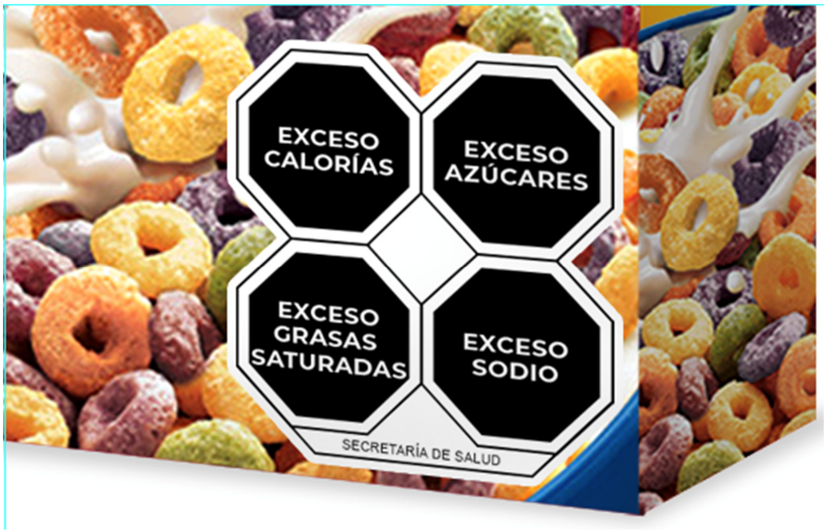

3c. Sugar Sweet Beverage

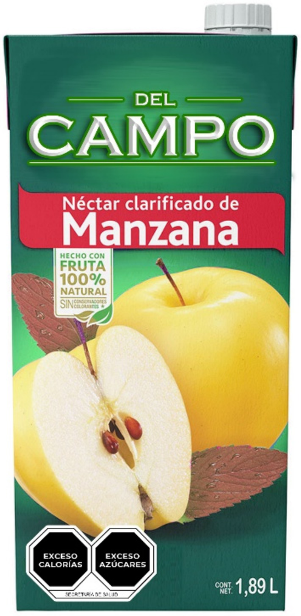

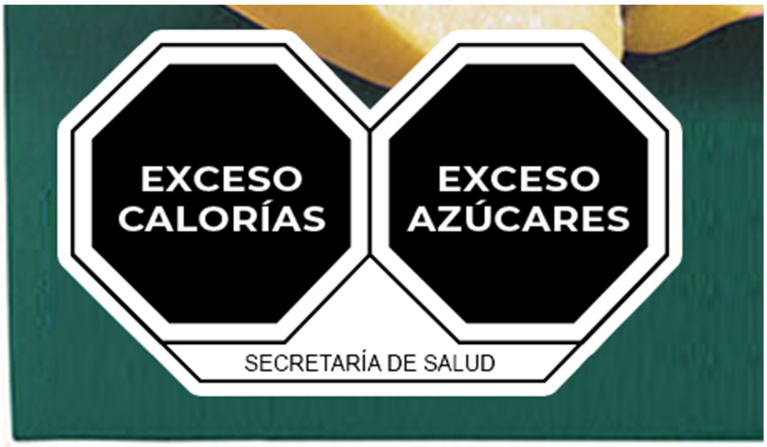

2d. Dairy

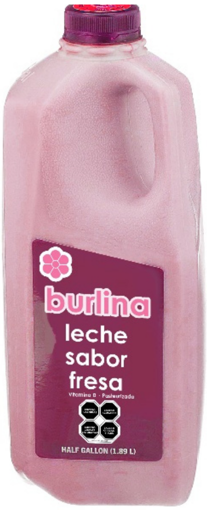

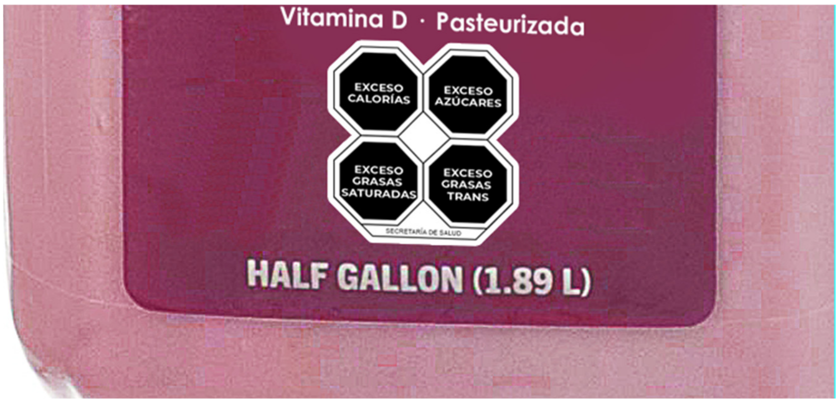

2e. Ready-to-eat Food

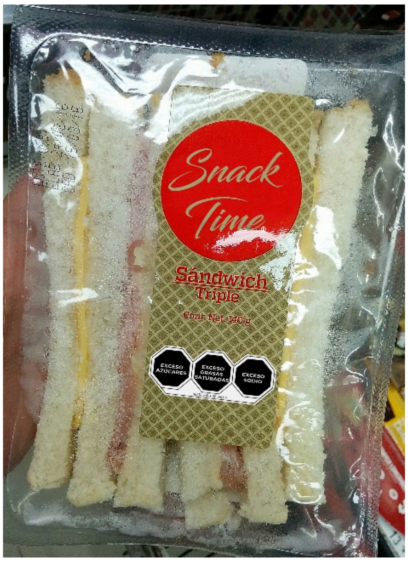

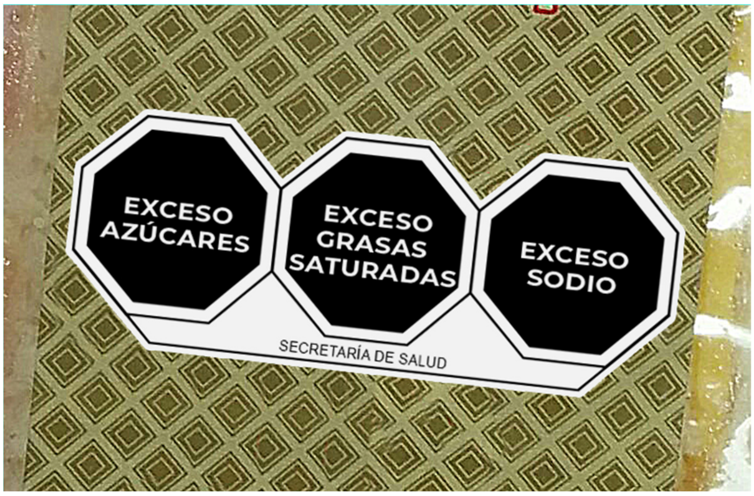

Supplement: Supplementary file 1 [file nutrients-14-03403-s001.zip › Figure S1. Products utilized in the study.pdf]
