# Supplementary material for: Perception and Understanding of Guideline Daily Amount and Warning Labeling among Mexican Adults during the Law Modification Period
Source: Nutrients. 2022 Aug 18;14(16):3403. doi: 10.3390/nu14163403 (PMC9415154; doi:10.3390/nu14163403)
Supplement: Supplementary file 1 [file nutrients-14-03403-s001.zip › File S1. Questionnaire used in both evaluations.pdf]

FULL QUESTIONNAIRE

Part 1. WELCOME

Dear participant

Hoping you are doing well, it is a pleasure to have your participation to complete this online survey of the project *"Potential results and effects of the implementation of Warning Labeling (NOM-051) in Mexican consumers".* Your answers in this study will be very valuable in food and nutrition policies in the country.

We appreciate your time in answering it.

Any questions or concerns in this regard do not hesitate to write to:

PhD. Ismael Campos, researcher responsible for the project
Mail. icampos@insp.mx
cPhD Jorge Vargas, co-investigator and general coordinator of the project
Mail. jorge.vargas@insp.mx

Receive a cordial greeting

[Informed consent must have been shown beforehand. Upon accepting the consent, the participant must fill out a socio-demographic and nutrition questionnaire prior to the label questions]

Part 2. GENERAL QUESTIONS AND NUTRITION QUESTIONNAIRE

Instructions. *Check the box according to the appropriate answer option:*

QUESTIONS	ANSWERS
**1. Sex**	☐ Feminine<br>☐ Male
**1.1 Sex of your child** (if applicable)	☐ Feminine<br>☐ Male
**2. Date of birth of the participating adult**	*[“DD/MM/YYYY”]*
**2.1 Date of Birth of the child**<br>(if apply)	*[“DD/MM/YYYY”]*
**3 What is your current weight ?**	*[Put option to put your weight in kilograms]*
**3 What is the height of you ?**	*[Put option to put your height in centimeters]*
**3.1 What is your child's current weight?**<br>(if apply)	*[Put option to put your weight in kilograms]*
**3.2 How tall is your child?** (if apply)	*[Put option to put your height in centimeters]*
**4. Marital Status**	☐ Single<br>☐ Married<br>☐ divorced / separated<br>☐ Free Union<br>☐ Widower
**5 Last level of education (last grade of studies you finished):**	☐ None<br>☐ Preschool<br>☐ Incomplete Primary<br>☐ Complete Primary<br>☐ Secondary incomplete<br>☐ Secondary complete<br>☐ Preparatory incomplete<br>☐ Preparatory complete<br>☐ Bachelor's degree incomplete<br>☐ Bachelor's degree complete<br>☐ Postgraduate<br>☐ Does not know

	☐ does not answer
**6. How many full bathrooms with shower and toilet (toilet) does your home have?**	☐ 0) 0 (None bath complete)<br>☐ 1) 1 Bathroom complete<br>☐ 2) 2 or more toilets complete<br>☐ 99) Don't know<br>☐ 88) No answer
**8. How many cars or trucks do you have in your household, including closed, cab, or box trucks?**	☐ 0) 0<br>☐ eleven<br>☐ 2) 2 or more<br>☐ 99) Don't know<br>☐ 88) No answer
**9. Without taking into account the mobile connection you could have from a cell phone, does your home have WIFI internet?**	☐ 0) Does not have<br>☐ 1) If you have<br>☐ 99) Don't know<br>☐ 88) No answer
**10. Of all the people aged 14 or over who live in the household, how many worked in the last month?**	☐ 0) 0<br>☐ eleven<br>☐ 2) 2<br>☐ 3) 3<br>☐ 4) 4 or more<br>☐ 99) Don't know<br>☐ 88) No answer
**11. In your home, how many rooms are used for sleeping, not counting hallways or bathrooms?**	☐ 0) 0<br>☐ eleven<br>☐ 2) 2<br>☐ 3) 3<br>☐ 4) 4 or more<br>☐ 99) Don't know<br>☐ 88) No answer
**12. Occupation**	☐ jobless _<br>☐ Student<br>☐ Home house<br>☐ Domestic<br>☐ Employee<br>☐ Businessman<br>☐ Other : ______________
**13 Has a doctor, nurse, or nutritionist told you that you suffer or have suffered from:**<br><br>*[Caption: "You can select more than one answer option"]*	☐ diabetes or sugar elevated<br>☐ Hypertension or pressure high<br>☐ Overweight<br>☐ Obesity<br>☐ high cholesterol<br>☐ high triglycerides
**14. Has a doctor, nurse, or nutritionist told you that your child suffers or has suffered from:**<br>(if apply)	☐ diabetes or sugar elevated<br>☐ Hypertension or pressure high<br>☐ Overweight

[Caption: "You can select more than one answer option"]	☐ Obesity<br>☐ high cholesterol<br>☐ high triglycerides
**15. Monthly household income**<br>(Over 18 years old inside the home)<br><br>*[Legend: "Amount in pesos"]*	*[Legend: "$________.Mexican pesos"]*
**16. How much do you think you know about nutrition?**	☐ I don't know anything about nutrition<br>☐ I only know a little about nutrition<br>☐ I know more or less about nutrition<br>☐ I know too much on nutrition
**17. How often do you shop for food or groceries for the household?**	☐ Never<br>☐ Almost never<br>☐ sometimes _<br>☐ Almost forever<br>☐ Always<br>☐ I don't know
**18. How often do you shop <u>online</u> for food or home groceries?**	☐ Never<br>☐ Almost never<br>☐ sometimes _<br>☐ Almost forever<br>☐ Always<br>☐ I don't know
**1 9. How much do you spend on average in a week on food or groceries for your household? What is the percentage of spending you spend on buying packaged food**	☐ Less than $300 pesos<br>☐ Between $300 and $500 pesos<br>☐ Between $500 and $1,000 pesos<br>☐ Between $1,000 and $1,500 pesos<br>☐ More than $1500 pesos<br>☐ I don't know<br>☐
**20. Do you use or have you used the nutritional front label of packaged foods and beverages?**	☐ Yes (go to question 21)<br>☐ No (continue)

20.1 Why do you not use nutrition labels on the packaging of packaged foods and bottled beverages?

0) You don't have time 1) You don't care 2) You can't see them 3) You don't trust them

4) Does not understand 5) Does not consider them useful 6) Other (Write here): _________

21. When you are deciding whether to buy a new food or drink, that is, something you have not bought before, what do you look <u>for</u> when deciding to buy it?

[More than one option can be selected]

1) In the brand or product name

2) In the price
3) In the characters on the packaging or promotions such as gifts or raffles
4) Product promotions (type 2 for 1, give away another product, etc.)
5) Because of the nutritional labeling that comes on the front of the wrapper (container)
6) For the information on the back of the wrapper (container) that describes the nutritional information (Table).
7) For the information on the back of the wrapper (container) that describes the ingredients.
8) In what your friends, relatives or friends of your child buy or consume
9) In that it is recommended by a health professional (nutritionist, doctor, other)
10) It is recommended by an influencer , blogger, youtuber , etc. (something I saw on social networks)
11) When you see it on TV
12) Other reasons (Write here): _________
99) Don't know/doesn't answer

22.1 Select the 3 most important (if applicable):

1.________________
two.________________
3.________________

Part 3. FRONT OF PACK LABELING SCHEME

[First Evaluation: GDA label. Second Evaluation: Warning label.]

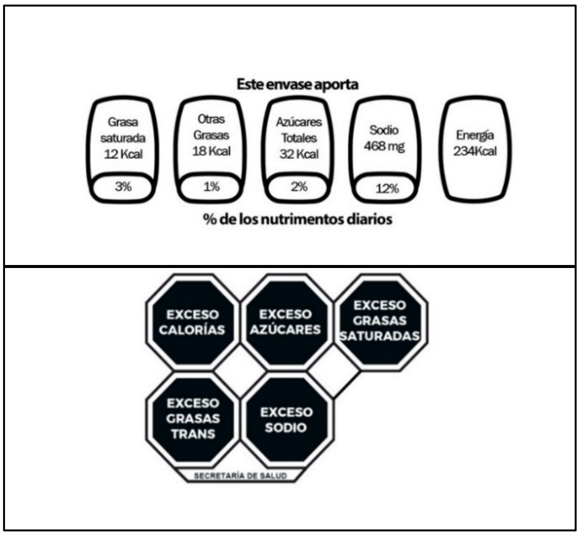

Instructions. *According to the picture of FRONT NUTRIENT LABELING shown, select the answer option that best corresponds to your opinion:*

4.1 In your opinion, how often should a product with this label be consumed?

Where 1 is not likely and 7 is very likely:

○	○	○	○	○	○	○
**1**	**2**	**3**	**4**	**5**	**6**	**7**
NO PROBABLE						MUY PROBABLE

4.2 In your opinion, in what quantities should a product with this label be consumed?
Where 1 is less and 7 if the amount is greater:

○	○	○	○	○	○	○
**1**	**2**	**3**	**4**	**5**	**6**	**7**
En pequeñas cantidades						En grandes cantidades

4.3 What would you do if you saw this label on a product you usually buy?
Where 1 is lower the probability of buying it and 7 you would continue to buy it:

○	○	○	○	○	○	○
**1**	**2**	**3**	**4**	**5**	**6**	**7**
No lo compraría						Continuaría comprándolo

4. 4 The product label catches my attention…

○	○	○	○	○	○	○
**1**	**2**	**3**	**4**	**5**	**6**	**7**
Totalmente en desacuerdo	Muy en desacuerdo	Algo en desacuerdo	Ni de acuerdo ni en desacuerdo	Algo de acuerdo	Muy de acuerdo	Totalmente de acuerdo

4.5 The product label is not visible

○ ○ ○ ○ ○ ○ ○

**1**	**2**	**3**	**4**	**5**	**6**	**7**
Totalmente en desacuerdo	Muy en desacuerdo	Algo en desacuerdo	Ni de acuerdo ni en desacuerdo	Algo de acuerdo	Muy de acuerdo	Totalmente de acuerdo

---

4.6 This label is easy to understand

○	○	○	○	○	○	○
**1**	**2**	**3**	**4**	**5**	**6**	**7**
Totalmente en desacuerdo	Muy en desacuerdo	Algo en desacuerdo	Ni de acuerdo ni en desacuerdo	Algo de acuerdo	Muy de acuerdo	Totalmente de acuerdo

---

4.7 This label will help me quickly decide which products to buy

○	○	○	○	○	○	○
**1**	**2**	**3**	**4**	**5**	**6**	**7**
Totalmente en desacuerdo	Muy en desacuerdo	Algo en desacuerdo	Ni de acuerdo ni en desacuerdo	Algo de acuerdo	Muy de acuerdo	Totalmente de acuerdo

---

4.8 I think this label will not help me identify healthier foods

○	○	○	○	○	○	○
**1**	**2**	**3**	**4**	**5**	**6**	**7**
Totalmente en desacuerdo	Muy en desacuerdo	Algo en desacuerdo	Ni de acuerdo ni en desacuerdo	Algo de acuerdo	Muy de acuerdo	Totalmente de acuerdo

---

4.9 This label will help me decide whether or not to buy a product

○	○	○	○	○	○	○
**1**	**2**	**3**	**4**	**5**	**6**	**7**
Totalmente en desacuerdo	Muy en desacuerdo	Algo en desacuerdo	Ni de acuerdo ni en desacuerdo	Algo de acuerdo	Muy de acuerdo	Totalmente de acuerdo

---

4.10 I believe that the information on this label is credible and true

○	○	○	○	○	○	○
**1**	**2**	**3**	**4**	**5**	**6**	**7**
Totalmente en desacuerdo	Muy en desacuerdo	Algo en desacuerdo	Ni de acuerdo ni en desacuerdo	Algo de acuerdo	Muy de acuerdo	Totalmente de acuerdo

4.11 This label will not change my decision about which products to buy

○	○	○	○	○	○	○
**1**	**2**	**3**	**4**	**5**	**6**	**7**
Totalmente en desacuerdo	Muy en desacuerdo	Algo en desacuerdo	Ni de acuerdo ni en desacuerdo	Algo de acuerdo	Muy de acuerdo	Totalmente de acuerdo

Part 4. QUESTIONS FOR PRODUCTS WITH FRONT OTF PACK LABELLING.
[Figure S1. First Evaluation: Five products with GDA label. Second Evaluation: Five products with Warning label.]

I. Instructions. The photo on the left side shows the front of a product. At the bottom of it, there is a front label that contains nutritional information.
II. The photo on the right shows the front labeling of this product.

According to the product and its front labeling, answer the following questions:

3.1 How attractive is the product for consumption?
Where 1 is unattractive and 7 is very attractive:

○	○	○	○	○	○	○
**1**	**2**	**3**	**4**	**5**	**6**	**7**
Poco atractivo						Muy atractivo

3.2 How healthy is the product?
Where 1 is unhealthy and 7 is very healthy:

○	○	○	○	○	○	○
**1**	**2**	**3**	**4**	**5**	**6**	**7**
No Saludable						Muy Saludable

3.3 Would you buy this product for yourself or your family?
Where 1 is not likely and 7 is very likely:

○	○	○	○	○	○	○
**1**	**2**	**3**	**4**	**5**	**6**	**7**
NO PROBABLE						MUY PROBABLE

3.4 How often would you buy this product for yourself or your child?

○	○	○	○	○	○	○
**1**	**2**	**3**	**4**	**5**	**6**	**7**
Casi nunca	Una vez al mes	Dos veces al mes	Una vez a la semana	Varias veces a la semana	Todos los días	Más de una vez al día

---

3.5 Does the front labeling of this product give you enough information to determine if it is healthy?

a) Not informative enough
b) It is informative
c) It is sufficiently informative
d) I don't know
e) Refused to answer

3.6 In general, does front labeling make you feel…?

a) LESS confident or able to decide if the product is healthy
b) NEITHER MORE NOR LESS sure or able to decide if the product is healthy
c) MORE confident or able to decide if the product is healthy
d) I don't know
e) Refuse to answer

3.7 In your opinion, in this product, which nutrient or nutrients are found in high amounts or higher than those recommended for a healthy diet? You can select more than one option

1) fats
2) sugars
3) Energy/ Calories
4) Sodium
5) None of these nutrients are high or above

[Questions for the Second Evaluation]

Part 5a. SWEETENERS DISCLAIMER

Instructions*.* *The next questions are about* ***sweeteners****. [Read: "A sweetener is a product used to sweeten foods and beverages in replacement or substitution of sugar. These have no calories and those that do have are very few compared to sugar. Some known ones are Stevia, splenda etc."].*
When packaged foods and beverages have added sweeteners, the following disclaimer appears:

CONTIENE EDULCORANTES, NO RECOMENDABLE EN NIÑOS

5.1 **What would you do if you saw this disclaimer on a product that you usually buy?**

○	○	○	○	○	○	○
**1**	**2**	**3**	**4**	**5**	**6**	**7**
No lo compraría						Continuaría comprándolo

5.2 **This disclaimer will help me decide whether or not to buy a product**

○ ○ ○ ○ ○ ○ ○

1	2	3	4	5	6	7
Totalmente en desacuerdo	Muy en desacuerdo	Algo en desacuerdo	Ni de acuerdo ni en desacuerdo	Algo de acuerdo	Muy de acuerdo	Totalmente de acuerdo

---

5.3 **How often would you give a product with this disclaimer to a child (if I had: my son or daughter)?**

○	○	○	○	○	○	○
**1**	**2**	**3**	**4**	**5**	**6**	**7**
Casi nunca	Una vez al mes	Dos veces al mes	Una vez a la semana	Varias veces a la semana	Todos los días	Más de una vez al día

---

5.4 **In your opinion, in what quantities should a product with this disclaimer be consumed?**
Where 1 is less and 7 if the amount is greater:

○	○	○	○	○	○	○
**1**	**2**	**3**	**4**	**5**	**6**	**7**
En pequeñas cantidades						En grandes cantidades

5.5 **How severe is the disclaimer that is in front of the product?**

a) Not severe enough
b) correct
c) too hard
d) I don't know
e) Refused to answer

Part 5b. CAFFEINE DISCLAIMER

Instructions. *Lastly, the next questions are about* **caffeine**. *[Read: "Caffeine is a bitter substance found naturally in different plants, such as coffee beans and tea leaves. Caffeine is primarily used in foods and beverages to flavor, fix flavor, as well as add color to foods.*
When packaged foods and beverages have caffeine in their ingredients, the following disclaimer appears:

CONTIENE CAFEÍNA EVITAR EN NIÑOS

6.1 **What would you do if you saw this disclaimer on a product that you usually buy?**

○	○	○	○	○	○	○
**1**	**2**	**3**	**4**	**5**	**6**	**7**
No lo compraría						Continuaría comprándolo

6.2 **This disclaimer will help me decide whether or not to buy a product**

○	○	○	○	○	○	○
**1**	**2**	**3**	**4**	**5**	**6**	**7**
Totalmente en desacuerdo	Muy en desacuerdo	Algo en desacuerdo	Ni de acuerdo ni en desacuerdo	Algo de acuerdo	Muy de acuerdo	Totalmente de acuerdo

---

6.3 **How often would you give a product with this disclaimer to a child (if I had: my son or daughter)?**

○	○	○	○	○	○	○
**1**	**2**	**3**	**4**	**5**	**6**	**7**
Casi nunca	Una vez al mes	Dos veces al mes	Una vez a la semana	Varias veces a la semana	Todos los días	Más de una vez al día

---

6.4 **In your opinion, in what quantities should a product with this disclaimer be consumed?**

Where 1 is less and 7 if the amount is greater:

○	○	○	○	○	○	○
**1**	**2**	**3**	**4**	**5**	**6**	**7**
En pequeñas cantidades						En grandes cantidades

6.5 **How severe is the disclaimer that is in front of the product?**

a) Not severe enough
b) correct
c) too hard
d) I don't know
e) Refused to answer

Part. 6. FINAL

We appreciate your collaboration in filling out this questionnaire. The information you provide us will be very valuable for the implementation of public policies that promote healthy eating. As I mentioned at the beginning, you will be sent infographics with recommendations from nutrition experts that will help you choose healthier packaged foods.
