# Supplementary material for: Perception and Understanding of Guideline Daily Amount and Warning Labeling among Mexican Adults during the Law Modification Period
Source: Nutrients. 2022 Aug 18;14(16):3403. doi: 10.3390/nu14163403 (PMC9415154; doi:10.3390/nu14163403)
Supplement: Supplementary file 1 [file nutrients-14-03403-s001.zip › Table S1. Change in perception and subjective understanding among GDA and WL by food category.pdf]

Table S1. Changes (%) in perception and subjective understanding among products with GDA and WL by food category[1].

	Dairy				Ready-to-eat cereal				Salty snack				Sugar Sweet Beverage				Ready-to-eat Food			
	GDA		WL		GDA		WL		GDA		WL		GDA		WL		GDA		WL	
**Perception**	%	95% CI	%	95% CI	%	95% CI	%	95% CI	%	95% CI	%	95% CI	%	95% CI	%	95% CI	%	95% CI	%	95% CI
How attractive is the product for consumption?[2] (Attractive or very attractive)	18.3	16.0, 20.6	13.8*	11.8, 15.9	39.0*	36.1, 41.8	17.9*	15.6, 20.2	36.8*	34.0, 39.7	22.3*	19.9, 24.8	41.6*	38.7, 44.6	22.6	20.1. 25.1	11.0*	9.1, 12.8	6.5*	5.0, 7.9
How healthy is the product?[3] (Healthy or very healthy)	13.2	11.2, 15.3	4.8*	3.5, 6.1	24.5*	21.9, 27.0	3.8*	2.7, 4.9	14.2*	12.2, 16.3	6.1*	4.7, 7.5	22.2*	19.7, 24.6	9.2	7.5, 10.9	9.1*	7.4, 10.8	4.0*	2.8, 5.1
Would you buy this product for yourself or your family?[2] (Unlikely-very unlikely)	11.9	10.0, 13.8	6.9*	5.4, 8.4	30.9*	28.2. 33.7	10.9*	9.0, 12.7	23.2*	20.7, 25.7	16.1*	13.9, 18.3	28.4*	25.7, 31.0	17.3	15.0, 19.5	9.0*	7.4, 10.7	4.8*	3.5, 6.1
How often would you buy this product for yourself?[2] (once or twice per month or never)	90.0	88.3, 91.9	94.0*	92.6, 95.4	88.9*	87.1, 90.8	94.7*	93.4, 96.1	85.0*	82.9, 87.1	88.3*	86.4, 90.2	74.4*	71.9, 77.0	80.4	78.1, 82.8	93.9*	92.4, 95.3	96.0*	94.9, 97.2
Does the label of this product provide enough information to determine if it´s healthy?[4] (Is not informative enough)	36.5	33.6, 39.3	14.3*	12.2, 16.4	34.5*	31.7, 37.3	12.1*	10.2, 14.0	27.1*	24.4, 29.7	11.8*	9.9, 13.7	28.1*	25.4, 30.7	13.3	11.3, 15.3	30.7*	28.0, 33.5	13.1*	11.1, 15.1
Front of pack labeling makes you feel?[4] (Safer to decide if the product is healthy)	27.5	24.9, 30.1	65.7*	62.8, 68.5	30.0*	27.3. 32.7	67.4*	64.6, 70.2	31.1*	28.4, 33.8	66.0*	63.2, 68.9	31.9*	29.2, 34.7	66.1	63.3, 68.9	28.3*	25.6, 30.9	65.3*	62.4, 68.1
**Subjective Understanding**																				
Correct identification of total number of high critical nutrients[5]	52.4	49.5, 55.4	91.5*	89.9, 93.2	13.6*	11.6, 15.6	88.5*	86.6, 90.4	35.2*	32.4, 38.0	90.0*	88.2, 91.8	68.0*	65.3, 70.8	95.8	94.6, 97.0	54.7*	51.7, 57.6	89.8*	87.9, 91.6

* Significant difference ($p < 0.05$) versus GDA.

GDA. Guideline Daily Allowance; WL, Warning Label.

The information presented refer differences in percentage (95%CI) of the population that respond to the category mentioned of each product after implementation of WL vs GDA.

[1]Adjusted percentages obtained through logistic regression models, as predictive variables were included age group, study stage and their interactions, besides, SES, nutrition knowledge and BMI, through predictive margins.

[2] Based on scales from 1 to 7 recoded to 2 categories. The categories presented represent the highest category of each variable.

[3] Based on scales from 1 to 7 recoded to 2 categories. The categories presented represent the lowest category of each variable.

[4] Based on a scale of 3 categories recoded to 2 categories. The category presented represent the highest category of each variable.

[5] Two categories variable. The category represents the correct identification of high amounts of fat, sugars, calories/energy and sodium.
