# Supplementary material for: Perception and Understanding of Guideline Daily Amount and Warning Labeling among Mexican Adults during the Law Modification Period
Source: Nutrients. 2022 Aug 18;14(16):3403. doi: 10.3390/nu14163403 (PMC9415154; doi:10.3390/nu14163403)
Supplement: Supplementary file 1 [file nutrients-14-03403-s001.zip › Table S2. Knowledge of sweetener∩s and perception of the sweetener and caffeine legends.pdf]

Table S2. Knowledge of sweetener´s and perception of the sweetener and caffeine disclaimers (n=1,083)

**Sweetener's disclaimer**	**%**	**95%CI**
Sweeteners are harmful to health (Strongly agree - totally agree)	32.3	29.6, 35.3
Many of the beverages, sweets, such as sodas and fruit nectars, have sweeteners (Strongly agree - totally agree)	52.3	49.3, 55.2
Sweeteners allow us to consume fewer calories (Strongly agree - totally agree)	31.9	29.2, 34.8
Boys and girls should not consume foods or drinks with sweeteners (Strongly agree - totally agree)	44.9	41.9, 47.9
Sweeteners are more harmful than sugar (Strongly agree - totally agree)	27.8	25.2, 30.5
Would you continue to buy a product that contains this disclamer? (Strongly disagree - totally disagree)	46.9	43.9, 49.9
This disclamer will help me decide whether or not to buy a product (Strongly agree - totally agree)	51.6	48.6, 54.6
How often would you give a product with this disclamer to a child (if you had a son or daughter)? (almost never)	73.6	70.9, 76.1
In your opinion, in what quantities should a product be consumed with this disclamer? (Very small quantities)	68.9	66.1, 71.6
How severe is the disclamer in front of the product		
Not enough	13.5	11.6, 15.7
Correct	60.8	57.8, 63.5
Too hard	13.6	11.7, 15.7
**Caffeine disclaimer**		
Would you continue to buy a product that contains this disclamer? (Strongly disagree - totally disagree)	42.9	40.0, 45.9
This disclamer will help me decide whether or not to buy a product (Strongly agree - totally agree)	47.4	44.4, 50.4
How often would you give a product with this disclamer to a child (if you had a son or daughter)? (almost never)	81.3	78.8, 83.5
In your opinion, in what quantities should a product be consumed with this disclamer? (Very small quantities)	65.1	62.2, 67.9
How severe is the disclamer in front of the product		
Not enough	12.9	11.1, 15.1
Correct	63.6	60.7, 66.4
Too hard	12.7	10.9, 14.9

Table S2a. Knowledge of sweetener´s and perception of the sweetener and caffeine disclamers according to population group and interest in health. (n=1083)

	Population group						Interest in own health					
	18 - 25 years n=382		26- 42 years n =329		43-75 years n=372		A little-Nothing n=34		Enough n=337		A lot n=712	
**Sweetener's**	%	95%CI	%	95%CI	%	95%CI	%	95%CI	%	95%CI	%	95%CI
Sweeteners are harmful to health (Strongly agree - totally agree)	28.5	24.2, 33.3	31.6	26.8, 36.8	36.8	32.1, 41.9	29.4	16.5, 46.9	30.9	26.1, 36.0	33.1	29.8, 36.7
Many of the beverages, sweets, such as sodas and fruit nectars, have sweeteners (Strongly agree - totally agree)	48.4	43.4, 53.5	52.3	46.9, 57.6	56.2	51.1, 61.2	50.0	33.6, 66.4	51.6	46.3, 56.9	52.7	49.0, 56.3
Sweeteners allow us to consume fewer calories (Strongly agree - totally agree)	28.5	24.2, 33.3	33.1	28.2, 38.4	34.4	29.7, 39.4	26.5	14.2, 43.8	26.7	22.2, 31.7	34.7	31.3, 38.3
Boys and girls should not consume foods or drinks with sweeteners (Strongly agree - totally agree)	36.1	31.4, 41.1	45.3	40.0, 50.7	53.5	48.4, 58.5	32.4	18.7, 49.8	41.8	36.7, 47.2	46.9	43.3, 50.6
Sweeteners are more harmful than sugar (Strongly agree - totally agree)	23.0	19.1, 27.5	29.5	24.8, 34.7	31.2	26.7, 36.1	29.4	16.5, 46.9	23.1	18.9, 28.0	29.9	26.7, 33.4
Would you continue to buy a product that contains this disclamer? (Strongly disagree - totally disagree)	43.7	38.8, 48.8	50.2	44.8, 55.5	47.3	42.3, 52.4	64.7	47.3, 78.9	42.1	37.0, 47.5	48.3	44.7, 52.0
This disclamer will help me decide whether or not to buy a product (Strongly agree - totally agree)	42.4	37.5, 47.4	56.8	51.4, 62.1	56.5	51.4, 61.4	61.8	44.4, 76.5	44.2	39.0, 49.6	54.6	51.0, 58.3
How often would you give a product with this disclamer to a child (if you had a son or daughter)? (almost never)	70.2	65.4, 74.5	73.9	68.8, 78.3	76.9	72.3, 80.9	70.6	53.1, 83.5	72.1	67.1, 76.6	74.4	71.1, 77.5
In your opinion, in what quantities should a product be consumed with this disclamer? (Very small quantities)	63.1	58.1, 67.8	73.6	68.5, 78.1	70.7	65.9, 75.1	76.5	59.2, 87.9	66.8	61.5, 71.6	69.5	66.0, 72.8
How severe is the disclamer in front of the product												
Not enough	15.4	12.2, 19.4	12.2	9.0, 16.2	12.6	9.6, 16.4	5.9	1.4, 21.1	13.4	10.1, 17.4	13.9	11.5, 16.7
Correct	56.5	51.5, 61.4	64.1	58.8, 69.1	62.1	57.0, 66.9	52.9	36.2, 69.0	58.8	53.4, 63.9	62.1	58.4, 65.6

Too hard	12.8	9.8,	16.6	13.1	9.8,	17.2	14.8	11.5,	18.8	26.5	14.2,	43.8	13.9	10.6,	18.1	12.8	10.5,	15.4
**Caffeine disclamer**																		
Would you continue to buy a product that contains this disclamer? (Strongly disagree - totally disagree)	37.7	33.0,	42.7	45.0	39.7,	50.4	46.5	41.5,	51.6	50.0	33.6,	66.4	40.4	35.2,	45.7	43.8	40.2,	47.5
This disclamer will help me decide whether or not to buy a product (Strongly agree - totally agree)	39.8	35.0,	44.8	52.0	46.6,	57.3	51.1	46.0,	56.1	55.9	38.9,	71.6	41.8	36.7,	47.2	49.6	45.9,	53.3
How often would you give a product with this disclamer to a child (if you had a son or daughter)? (almost never)	78.3	73.8,	82.1	83.9	79.5,	87.5	82.0	77.7,	85.6	79.4	62.4,	90.0	80.7	76.1,	84.6	81.6	78.6,	84.3
In your opinion, in what quantities should a product be consumed with this disclamer? (Very small quantities)	58.6	53.6,	63.5	69.3	64.1,	74.1	68.0	63.1,	72.6	79.4	62.4,	90.0	62.6	57.3,	67.6	65.6	62.0,	69.0
How severe is the disclamer in front of the product																		
Not enough	12.8	9.8,	16.6	12.8	9.6,	16.8	13.2	10.1,	17.0	8.8	2.8,	24.4	12.8	9.6,	16.8	13.2	10.9,	15.9
Correct	63.6	58.7,	68.3	65.7	60.3,	70.6	61.8	56.8,	66.6	58.8	41.6,	74.1	63.2	57.9,	68.2	64.0	60.4,	67.5
Too hard	11.8	8.9,	15.4	10.6	7.7,	14.5	15.6	12.2,	19.7	17.6	8.0,	34.4	13.4	10.1,	17.4	12.2	10.0,	14.8

Table S2b. Knowledge of sweetener´s and perception of the sweetener and caffeine disclamers according to academic level. (n=1083)

	Academic level					
	Secondary or lower n=169		High school n=359		Bachelor's degree or above n=555	
**Sweetener´s**	**%**	**95%CI**	**%**	**95%CI**	**%**	**95%CI**
Sweeteners are harmful to health (Strongly agree - totally agree)	30.8	24.3, 38.2	32.6	27.9, 37.6	32.6	28.8, 36.6
Many of the beverages, sweets, such as sodas and fruit nectars, have sweeteners (Strongly agree - totally agree)	44.4	37.0, 52.0	51.8	46.6, 57.0	55.0	50.8, 59.1
Sweeteners allow us to consume fewer calories (Strongly agree - totally agree)	33.1	26.4, 40.6	29.5	25.0, 34.5	33.2	29.4, 37.2
Boys and girls should not consume foods or drinks with sweeteners (Strongly agree - totally agree)	40.8	33.6, 48.4	43.5	38.4, 48.6	47.0	42.9, 51.2
Sweeteners are more harmful than sugar (Strongly agree - totally agree)	25.4	19.4, 32.6	26.7	22.4, 31.6	29.2	25.5, 33.1
Would you continue to buy a product that contains this disclamer? (Strongly disagree - totally disagree)	47.9	40.5, 55.5	47.6	42.5, 52.8	46.1	42.0, 50.3
This disclamer will help me decide whether or not to buy a product (Strongly agree - totally agree)	55.0	47.4, 62.4	51.5	46.4, 56.7	50.6	46.5, 54.8
How often would you give a product with this disclamer to a child (if you had a son or daughter)? (almost never)	68.0	60.6, 74.7	72.1	67.3, 76.5	76.2	72.5, 79.6
In your opinion, in what quantities should a product be consumed with this disclamer? Very small quantities	68.0	60.6, 74.7	68.8	63.8, 73.4	69.2	65.2, 72.9
How severe is the disclamer in front of the product						
Not enough	14.8	10.2, 21.0	10.6	7.8, 14.2	15.0	12.2, 18.2
Correct	53.3	45.7, 60.7	62.1	57.0, 67.0	62.2	58.0, 66.1

Too hard	16.0	11.2,	22.3	14.8	11.4,	18.8	12.1	9.6, 15.1
**Caffeine disclamer**								
Would you continue to buy a product that contains this disclamer? (Strongly disagree - totally disagree)	51.5	43.9,	59.0	41.2	36.2,	46.4	41.4	37.4, 45.6
This disclamer will help me decide whether or not to buy a product (Strongly agree - totally agree)	49.1	41.6,	56.6	47.9	42.8,	53.1	46.5	42.4, 50.7
How often would you give a product with this disclamer to a child (if you had a son or daughter)? (almost never)	78.1	71.2,	83.7	78.6	74.0,	82.5	84.0	80.7, 86.8
In your opinion, in what quantities should a product be consumed with this disclamer? (Very small quantities)	71.0	63.7,	77.4	62.4	57.3,	67.3	65.0	61.0, 68.9
How severe is the disclamer in front of the product								
Not enough	11.8	7.7,	17.7	10.6	7.8,	14.2	14.8	12.1, 18.0
Correct	56.2	48.6,	63.5	63.8	58.7,	68.6	65.8	61.7, 69.6
Too hard	18.9	13.7,	25.6	13.9	10.7,	17.9	10.1	7.8, 12.9

Table S2c. Knowledge of sweetener´s and perception of the sweetener and caffeine disclamers according to nutrition knowledge. (n=1083)

	Nutrition knowledge					
	More or less - too much n=433		A little n=505		Nothing n=145	
**Sweetener´s**	%	95%CI	%	95%CI	%	95%CI
Sweeteners are harmful to health (Strongly agree - totally agree)	33.7	29.4, 38.3	28.7	24.9, 32.8	40.7	33.0, 48.9
Many of the beverages, sweets, such as sodas and fruit nectars, have sweeteners (Strongly agree - totally agree)	57.3	52.6, 61.9	49.3	45.0, 53.7	47.6	39.6, 55.7
Sweeteners allow us to consume fewer calories (Strongly agree - totally agree)	34.6	30.3, 39.3	28.1	24.4, 32.2	37.2	29.7, 45.4
Boys and girls should not consume foods or drinks with sweeteners (Strongly agree - totally agree)	46.7	42.0, 51.4	42.6	38.3, 46.9	47.6	39.6, 55.7
Sweeteners are more harmful than sugar (Strongly agree - totally agree)	28.2	24.1, 32.6	25.5	21.9, 29.5	34.5	27.2, 42.6
Would you continue to buy a product that contains this disclamer? (Strongly disagree - totally disagree)	46.3	41.6, 51.0	45.3	41.0, 49.6	54.5	46.3, 62.4
This disclamer will help me decide whether or not to buy a product (Strongly agree - totally agree)	51.2	46.4, 55.9	53.2	48.8, 57.5	47.6	39.6, 55.7
How often would you give a product with this disclamer to a child (if you had a son or daughter)? (almost never)	75.0	70.7, 78.9	73.7	69.7, 77.4	69.0	60.9, 76.0
In your opinion, in what quantities should a product be consumed with this disclamer? Very small quantities	67.8	63.3, 72.1	69.2	65.0, 73.0	71.0	63.1, 77.9
How severe is the disclamer in front of the product						
Not enough	14.6	11.6, 18.2	10.9	8.4, 13.9	19.3	13.7, 26.6

Correct	64.6	59.9,	69.0	59.9	55.5,	64.1	52.4	44.3,	60.4
Too hard	10.0	7.5,	13.2	15.4	12.5,	18.8	17.9	12.5,	25.1
**Caffeine disclamer**									
Would you continue to buy a product that contains this disclamer? (Strongly disagree - totally disagree)	40.5	36.0,	45.2	41.1	36.9,	45.5	56.6	48.3,	64.4
This disclamer will help me decide whether or not to buy a product (Strongly agree - totally agree)	46.3	41.6,	51.0	48.8	44.5,	53.2	45.5	37.6,	53.7
How often would you give a product with this disclamer to a child (if you had a son or daughter)? (almost never)	82.6	78.8,	85.9	81.6	78.0,	84.8	75.9	68.2,	82.2
In your opinion, in what quantities should a product be consumed with this disclamer? (Very small quantities)	64.1	59.5,	68.5	63.8	59.5,	67.9	72.4	64.5,	79.1
How severe is the disclamer in front of the product									
Not enough	15.0	12.0,	18.7	10.5	8.1,	13.5	15.2	10.2,	22.0
Correct	65.5	60.9,	69.9	64.0	59.7,	68.1	56.6	48.3,	64.4
Too hard	9.7	7.3,	12.9	13.6	10.9,	16.9	18.6	13.1,	25.8
